# Ongoing evolution of PE/PPE genes in *Mycobacterium tuberculosis* associated with drug resistance and host immune response

**DOI:** 10.1128/msystems.00898-25

**Published:** 2025-09-22

**Authors:** Mingyu Gan, Dan Wang, Suqing Li, Qinglan Wang, Qingyun Liu

**Affiliations:** 1 Department of Genetics, University of North Carolina at Chapel Hill 2331, Chapel Hill, North Carolina, USA; 2 Center for Molecular Medicine, Children’s Hospital of Fudan University, National Children’s Medical Center, Shangha, China; 3 Department of Respiratory and Critical Care Medicine, Frontiers Science Center for Disease-related Molecular Network, Institute of Respiratory Health, West China Hospital, Sichuan University 12530, Chengdu, Sichuan, China; 4 Department of Microbiology and Immunology, University of North Carolina at Chapel Hill 2331, Chapel Hill, North Carolina, USA; Institut de Recherche pour le Developpement Delegation Regionale Occitanie, Montpellier, France

**Keywords:** *Mycobacterium tuberculosis*, PE/PPE, PPE51, drug resistance evolution

## Abstract

The Proline-Glutamate/Proline-Proline-Glutamate (PE/PPE) gene family comprises approximately 10% of the *Mycobacterium tuberculosis* (Mtb) genome and is characterized by GC-rich, highly repetitive sequences. As a result, these genes are usually excluded from short-read-based whole-genome sequencing analyses, leaving their sequence diversity and evolutionary dynamics poorly characterized. Recently, a genome masking approach demonstrated that roughly 54% of PE/PPE sequences are recoverable from short-read data, providing an opportunity to examine the evolution of this gene family at a population level. Here, we analyzed 51,229 Mtb genomes to characterize sequence diversity and selection pressures across the PE/PPE gene family. Overall, we observed that PE/PPE genes are under relaxed purifying selection compared to other gene categories, as evidenced by higher ratios of nonsynonymous to synonymous polymorphisms (*pNpS*) and greater mutation burdens. We identified 12 PE/PPE genes with signatures of positive selection and 7 with selective pressure associated with antibiotic resistance. Among these genes, *PPE51* exhibited selection favoring loss-of-function mutations, which occurred only in Mtb strains that were already multidrug-resistant (MDR). This pattern suggests either compensatory evolution or adaptation related to resistance against second-line or newly introduced drugs. Additionally, we identified T-cell epitopes in six PE/PPE genes that were subject to diversifying selection, suggesting immune-driven adaptation. Collectively, this work provides a baseline characterization of genetic diversity in PE/PPE genes and highlights specific genes that may be involved in adaptation to host immunity and antibiotic pressure and represent candidates for further investigation.

**IMPORTANCE:**

Tuberculosis remains a significant global health challenge, partly due to *Mycobacterium tuberculosis* (Mtb)’s remarkable evolutionary adaptation to antibiotics and human immune responses. Around 10% of its genome comprises PE/PPE genes, whose functions and evolutionary dynamics are poorly understood due to their repetitive sequences and high GC content. In this study, we analyzed 51,229 global Mtb genomes using an advanced genome-masking method, revealing numerous PE/PPE genes under positive selection, potentially facilitating antibiotic resistance and immune evasion. Notably, *PPE51* often loses its function in strains resistant to multiple antibiotics, suggesting a role in bacterial survival during drug treatment. Additionally, we identified mutation-prone regions within six PE/PPE genes, highlighting potential targets for future vaccine development. Collectively, our findings underscore the crucial role of PE/PPE genes in Mtb evolution and drug resistance, providing valuable insights to inform novel therapeutic and vaccine strategies.

## INTRODUCTION

Approximately 10% of the *Mycobacterium tuberculosis* (Mtb) genome encodes genes belonging to the PE/PPE family, comprising a total of 168 genes (69 PPE and 99 PE). PE and PPE genes are characterized by their conserved N-terminal sequences, known as the PE (Proline–Glutamate) and PPE (Proline–Proline–Glutamate) domains, respectively, which form a unique gene category within the Mtb genome. Since evolving from its most recent common ancestor, Mtb has lost the capacity for horizontal gene transfer, and the number of PE/PPE genes has remained consistent across different members of the Mtb complex (1).

A wide range of diverse functions has been attributed to the large family of Mtb PE/PPE genes (2). A recent study suggested that some PPEs and PEs, such as PPE51 and PE19, may form a porin-like protein complex involved in nutrient acquisition (3). Additionally, it was suggested that PE/PPE proteins can modulate host immune responses and cell death during infection. For example, PE-PGRS11 and PE-PGRS17 can interact with the host TLR2 receptor to induce dendritic cell maturation, thereby enhancing CD4+ T-cell proliferation and cytokine secretion (4). Similarly, PPE57 can activate macrophages via TLR2 to promote Th1 immune responses and increase interferon-γ and interleukin-2 production (5). Because many PE/PPE proteins are surface-exposed, secreted, and highly conserved, they have also been a key focus in TB vaccine development (2, 6). Vaccine candidate M72/AS01E incorporates fusion protein M72, which combines PPE18 (Rv1196) with non-PE/PPE protein Rv0125, and vaccine candidate ID93 contains PPE42 (Rv2608) as a core immunogenic component (2, 7).

The PE/PPE gene family has been implicated in biological processes or cellular components linked to mechanisms of antibiotic action, suggesting that Mtb may have evolved PE/PPE genes to counteract antibiotic stress. PE/PPE genes have also been associated with antibiotic resistance. For example, genetic variations in PPE54 were linked to resistance to rifampicin, isoniazid, and ethambutol (8), and mutations in PE_PGRS7 have been associated with resistance to streptomycin (9). PE11 increases antibiotic resistance by altering the lipid composition of the mycobacterial cell wall, thereby enhancing its hydrophobicity and decreasing antibiotic permeability (10).

The sequence evolution of PE/PPE genes in the global Mtb population remains poorly documented, largely because their high GC content, which can reach 80%, and their extensive sequence similarity and repetitive sequences complicate accurate analysis based on short-read sequencing data (11). As a result, these genes are typically excluded from analyses of short-read genomic sequencing, which has severely limited our understanding of their sequence variations (12, 13). Recently, however, Marin et al. proposed a genome masking scheme for removing genomic regions of Mtb from short-read sequencing data using long-read sequencing as a benchmark (14). Using this genome-masking scheme, approximately 54% of the PE/PPE sequences could be reliably analyzed from short-read sequencing data. This masking scheme makes it possible to use existing Mtb whole-genome, short-read sequences to investigate the diversity and evolution of PE/PPE genes. In this work, we characterize sequence variation and adaptive evolution in the PE/PPE gene family in 51,229 Mtb isolates by focusing on the 54% of the PE/PPE gene regions with high-quality sequencing data obtained using the masking scheme. We found that the PE/PPE gene family is predominantly under purifying selection, although a subset exhibits signatures of adaptive evolution. Within this subset, some genes are associated with antibiotic resistance, while others appear driven by host immune selection.

## RESULTS

### Mtb genome sequences and mutations in PE/PPE genes

**Fig 1:**
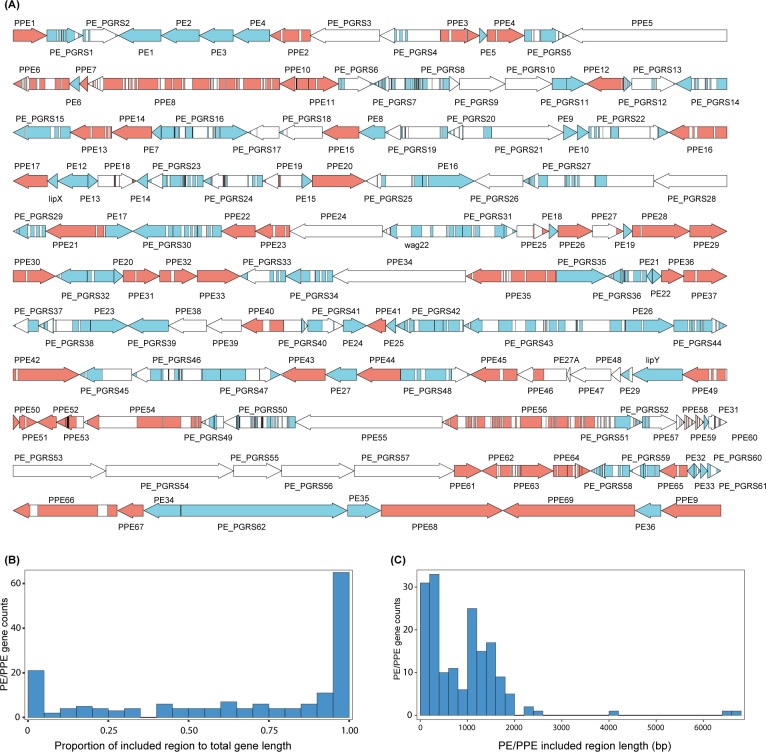
Coverage of analyzable regions in PE/PPE genes. (**A**) Schematic representation of high-quality analyzable regions within PE (blue) and PPE (red) genes. Genes are ordered according to their genomic positions, with white segments representing low-confidence regions not reliably analyzable by short-read sequencing. (**B**) Distribution showing the percentage of analyzable regions among 168 PE/PPE genes. (**C**) Distribution of lengths (in base pairs) of the analyzable regions across the PE/PPE genes.

In a recent study, we curated whole-genome sequencing (WGS) data from 51,229 Mtb strains isolated in 106 countries (15). After applying filtering criteria (Materials and Methods), 49,954 Mtb isolates were included in the current study (Table S1). Using the genome-masking scheme recently proposed by Marin et al. (14), we found that 31.5% (53/168) of the PE/PPE genes were fully covered in high-quality sequence regions, and an additional 19.6% (33/168) had >80% gene length covered by high-quality sequence (Fig. 1A and B; Tables S2 and S3). The coverage of high-quality regions varies across different gene categories, with PE_PGRS genes being particularly underrepresented. Specifically, only 46.8% (29/62) of PE_PGRS genes exhibit higher than 50% coverage, compared to 94.5% (35/37) of PE genes and 75.4% (52/69) of PPE genes. Additionally, the distribution of high-quality regions differs significantly between the PE/PPE domains and the C-terminus (Fig. S1). We observed that the proportion of high-quality coverage was notably higher in the PE/PPE domains compared to the C-terminus (*P*-value < 0.01). The total length of high-quality sequence across all PE/PPE genes was 151, 798 bp, representing 54% of their combined genomic length (281, 073 bp) (Fig. 1C). Within these regions, we identified 40,193 unique mutations, averaging 26.5 SNPs per 100 bp.

To identify the mutational events in PE/PPE genes, we used phylogenetic reconstruction to infer the mutational trajectories and merged all mutations affecting strains in the same phylogenetic clade into a single mutational event (Methods). To reduce the computational load, we divided the 49,954 isolates into 25 groups based on a previous sublineage-defining scheme and used phylogenetic proximity to balance the sample numbers in the different groups (16). Phylogenetic trees were then reconstructed separately for each group using the Maximum Likelihood Method incorporated in IQ-TREE2 (17), and mutation events were identified based on the mutational trajectory reconstruction in each tree. In total, we identified 53,843 mutational events in the 168 PE/PPE genes.

### Natural selective pressure on PE/PPE genes

PE/PPE genes have undergone expansion within slow-growing mycobacteria, perhaps as a compensatory mechanism in response to the loss of porin proteins in these mycobacteria (18). We posited that the selective pressures on these recently expanded genes could be different from pressures on the rest of the genome. Using *pNpS* and mutation burden as proxies for selective pressures, we analyzed 116 PE/PPE genes with at least 50% of their gene lengths covered by high-quality sequence. *pNpS* calculates the ratio of nonsynonymous (NS) to synonymous mutations (SY), normalized by the expected NS and SY mutations under neutral simulation, and a value greater than 1 suggests positive selection (19). The mutation burden calculates the normalized mutation events in a gene, adjusted for gene length. The mutation burden for PE/PPE genes was significantly higher than for both the essential and nonessential categories (Fig. S2A). Consistent with previous studies (20, 21), essential genes in the Mtb genome had significantly lower *pNpS* values than nonessential genes (0.69 vs. 0.84) (Fig. S2B). However, we found that the *pNpS* value for the PE/PPE family genes was significantly higher than for both the essential and nonessential genes in the rest of the genome (0.90 vs 0.69 and 0.84, *P* < 0.001) (Fig. S2B). We next divided the Mtb genes into different functional groups following the categories defined by Cole et al. (22) and found that the PE/PPE group showed significantly higher *pNpS* values and mutation burdens (*P* < 0.05, Wilcoxon test) than genes in the other categories (Fig. S2C and D). Together, these results showed that the PE/PPE genes exhibit significantly higher levels of sequence variation—both in mutation burden and *pNpS* ratio—compared to essential, nonessential, and other functional gene categories in the Mtb genome.

**Fig 2:**
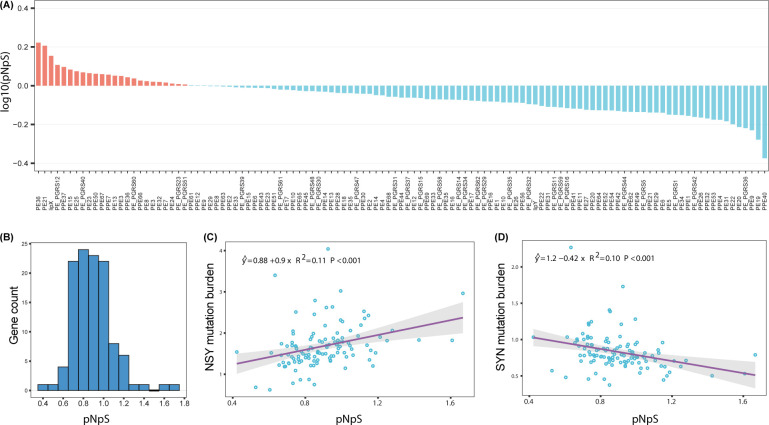
Distribution and correlation of *pNpS* values across PE/PPE genes. (**A**) Log₁₀-transformed *pNpS* values of PE/PPE genes ranked from highest to lowest. Genes with *pNpS* values greater than 1 are colored red, indicating positive selection, while genes with *pNpS* values less than 1 are colored blue, indicating purifying or neutral selection. (**B**) Histogram showing the distribution of *pNpS* values among PE/PPE genes. (**C**) Positive linear correlation between *pNpS* values and nonsynonymous mutation burden across PE/PPE genes. (**D**) Negative linear correlation between *pNpS* values and synonymous mutation burden across PE/PPE genes.

The increased sequence diversity in PE/PPE genes suggested that some of these genes may have experienced adaptive evolution. We next sought to identify positive selection signals in genes with a high-quality sequence coverage of >80% of gene length. In total, we found 12 genes with *pNpS* values suggesting positive selection (Fig. 2A and B, Table 1). For example, PE36 had a *pNpS* value of 1.67, with 105 NS mutation events and 28 SY mutation events, affecting a total of 598 isolates (Table 1). Similarly, *lipX* had a *pNpS* of 1.43 (NS:SY = 84:23), affecting 584 isolates. Interestingly, we observed premature stop mutations in codons 78, 88, and 92 of *lipX*, each occurring three times independently. In contrast, six PE/PPE genes had very low *pNpS* values, suggesting purifying selection to keep these genes conserved (Fig. 2A and B). *PPE40*, for example, had a *pNpS* value of 0.42, with 165 NS mutations and 167 SY mutations.

**TABLE 1:** Positively selected PE/PPE genes

Gene	Gene length	Included ratio	NS events	SY events	NS isolates	SY isolates	pNpS	*pNpS* Ds^*a*^	*pNpS* Dr^*a*^
PE36	234	1	105	28	537	61	1.67	1.44	1.85
lipX	303	1	84	23	362	222	1.43	1.80	1.28
PPE37	1,422	0.95	367	130	5,900	3,512	1.25	1.34	1.00
PE15	309	1	89	33	3,999	113	1.21	0.87	1.53
PE25	300	1	69	25	199	110	1.19	1.86	1.13
PE23	1,149	1	275	109	7,535	386	1.16	1.02	1.62
PE13	300	1	107	37	285	144	1.13	1.24	1.26
PPE3	1,611	0.90	550	187	11,461	738	1.12	1.07	0.95
PPE36	732	1	243	79	1,072	646	1.11	1.03	1.23
PE_PGRS60	315	1	92	37	2,113	66	1.09	1.02	0.93
PPE66	948	0.83	170	74	5,093	561	1.06	1.04	1.07
PE8	828	0.94	194	80	1,025	934	1.05	1.19	0.98

^*a*^ pNpS Ds, pNpS of drug susceptible isolates; pNpS Dr, pNpS of drug resistance isolates.

We posited that the selective pressures suggested by high *pNpS* values would positively correlate with mutation burden, as a gene under adaptive evolution would be expected to show not only a higher ratio of nonsynonymous to synonymous mutations but also a larger number of normalized nonsynonymous mutational events. To test this, we performed a linear regression that revealed a significant positive correlation (R^2^ = 0.11, *P* < 0.001) between *pNpS* values and the mutational burden for nonsynonymous mutations. To test whether the elevated mutational burden was due to an intrinsically higher mutation rate in these genes, we assessed the correlation using only synonymous mutations and found a negative correlation with *pNpS* (R^2^ = 0.1, *P* < 0.001) (Fig. 2C and D). The positive correlation between *pNpS* and the burden of nonsynonymous mutations is consistent with adaptive evolution in certain genes, while the negative correlation with the burden of synonymous mutations suggests that these patterns cannot be attributed solely to the intrinsic mutation rate.

### Evolution of PE/PPE genes under antibiotics pressure

**Fig 3:**
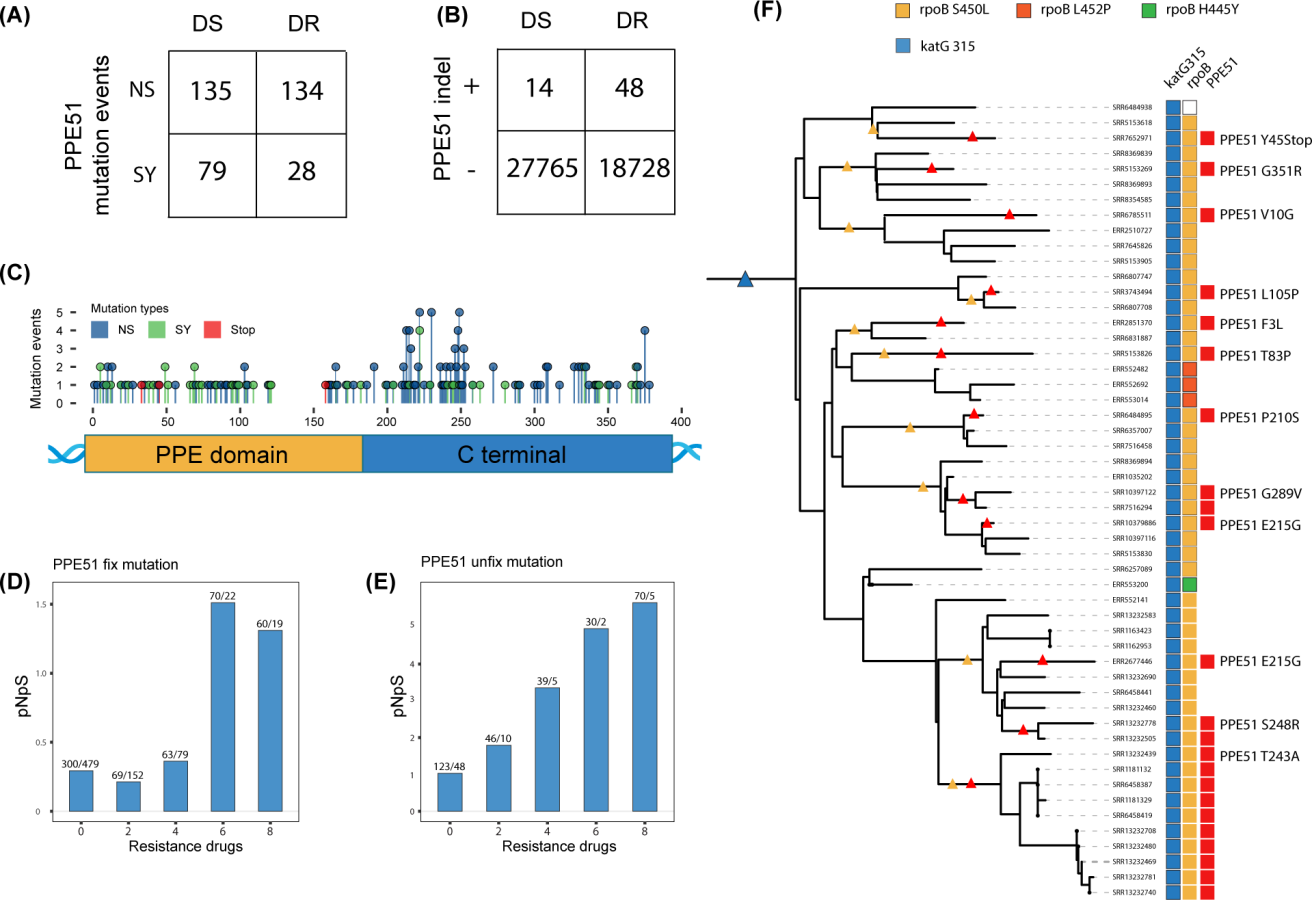
Association between PPE51 and multidrug resistance . (**A**) Significant enrichment of PPE51 nonsynonymous mutants in drug-resistant strains compared to drug-sensitive strains. (**B**) Significant enrichment of indel mutations in PPE51 among drug-resistant and drug-sensitive strains. (**C**) Distribution of mutation types and number of mutational events mapped along the PPE51 gene. (**D**) The *pNpS* ratio for PPE51 increases in strains resistant to 6 or more drugs. (**E**) Similar trends were observed in unfixed mutations, indicating active ongoing selection in drug-resistant strains. (**F**) Phylogenetic analysis illustrating sequential mutation events in PPE51 occurring after the emergence of resistance mutations in katG and rpoB. Twelve PPE51 mutation events are identified within a subclade of lineage 2.2.1, all of which are drug resistant. The blue triangle marks the phylogenetic position of the katG S315 mutation, yellow triangles indicate rpoB resistance mutations, and red triangles denote PPE51 mutation events.

To date, 17 genes have been validated to be associated with phenotypic drug resistance in Mtb (23), but none of these genes belong to the PE/PEE gene families. We reasoned that if any PE/PPE genes are involved in the evolution of drug resistance, mutations in these genes will be enriched in drug-resistant isolates. To search for PE/PPE genes that are potentially associated with drug resistance, we compared the *pNpS* ratios of each gene in drug-resistant and drug-sensitive strains and identified seven genes with significantly higher *pNpS* ratios in the drug-resistant group (Table 2). Among these genes, *PPE51* was of particular interest, with a *pNpS* value increasing from 0.8 in drug-sensitive strains to 2.08 in drug-resistant strains. In addition, we also found that the *PPE51* mutants were significantly enriched in drug-resistant strains (OR: 1.92, 95% CI: 1.29–2.87) (Fig. 3A). These *PPE51* mutations exhibited a hotspot between codons 210 and 250, where protein structural modeling predicted an α-helix-loop, suggesting that these mutations would have functional implications (Fig. 3C). Three of these mutations were premature stop codons that likely resulted in loss of *PPE51* function (Fig. 3C). Additionally, we analyzed insertions and deletions in *PPE51* across drug-sensitive and drug-resistant strains and found a significant enrichment of indels in the drug-resistant strains (OR: 5.08, 95% CI: 2.76–9.99) (Fig. 3B), 89.6% of which introduced frameshifts, further indicating a tendency for loss of *PPE51* function in drug-resistant strains.

**TABLE 2:** Drug resistance associated PE/PPE genes

Gene	Susceptible					Resistance				
	NS events	SY events	NS isolates	SY isolates	*pNpS*	NS events	SY events	NS isolates	SY isolates	*pNpS*
PPE51	135	79	205	188	0.80	134	28	286	69	2.08
PPE13	187	99	580	184	0.79	84	22	138	42	1.57
PE15	47	23	99	32	0.87	26	8	36	36	1.53
PE20	39	28	90	73	0.58	23	8	971	22	1.21
PE23	158	72	475	197	1.02	85	24	272	47	1.62
PE7	46	23	142	34	0.93	27	8	61	11	1.43
PPE10	249	129	623	255	0.81	110	37	227	70	1.26

Because Mtb evolves multi-drug resistance in a stepwise manner (24), we used a stepwise approach to infer the stage at which the *PPE51* mutations were selected. We grouped the isolates based on the number of drugs to which they were resistant and found that in isolates that were drug susceptible or resistant to two or four drugs, the *pNpS* ratio for *PPE51* remained below 1, suggesting purifying selection. In samples resistant to six or more drugs, however, the *pNpS* ratio increased to 1.5, suggesting positive selection (Fig. 3D). A similar trend was observed in the analysis of unfixed mutations, with the *pNpS* ratio rising as the number of resistant drugs increased (Fig. 3E). NS mutations in *PPE51* occurred independently 23 times in a subclade of lineage 2.2.1 drug-resistant strains, all of which were acquired after mutations in *katG* and *rpoB*, which confer resistance to isoniazid and rifampicin, respectively, and together constitute MDR-TB (Fig. 3F). The loss of *PPE51* function after strains have become MDR-TB suggests either compensatory evolution or selection by second-line, or perhaps new, recently introduced drugs.

### Genetic diversity in PE/PPE epitope regions

**Fig 4:**
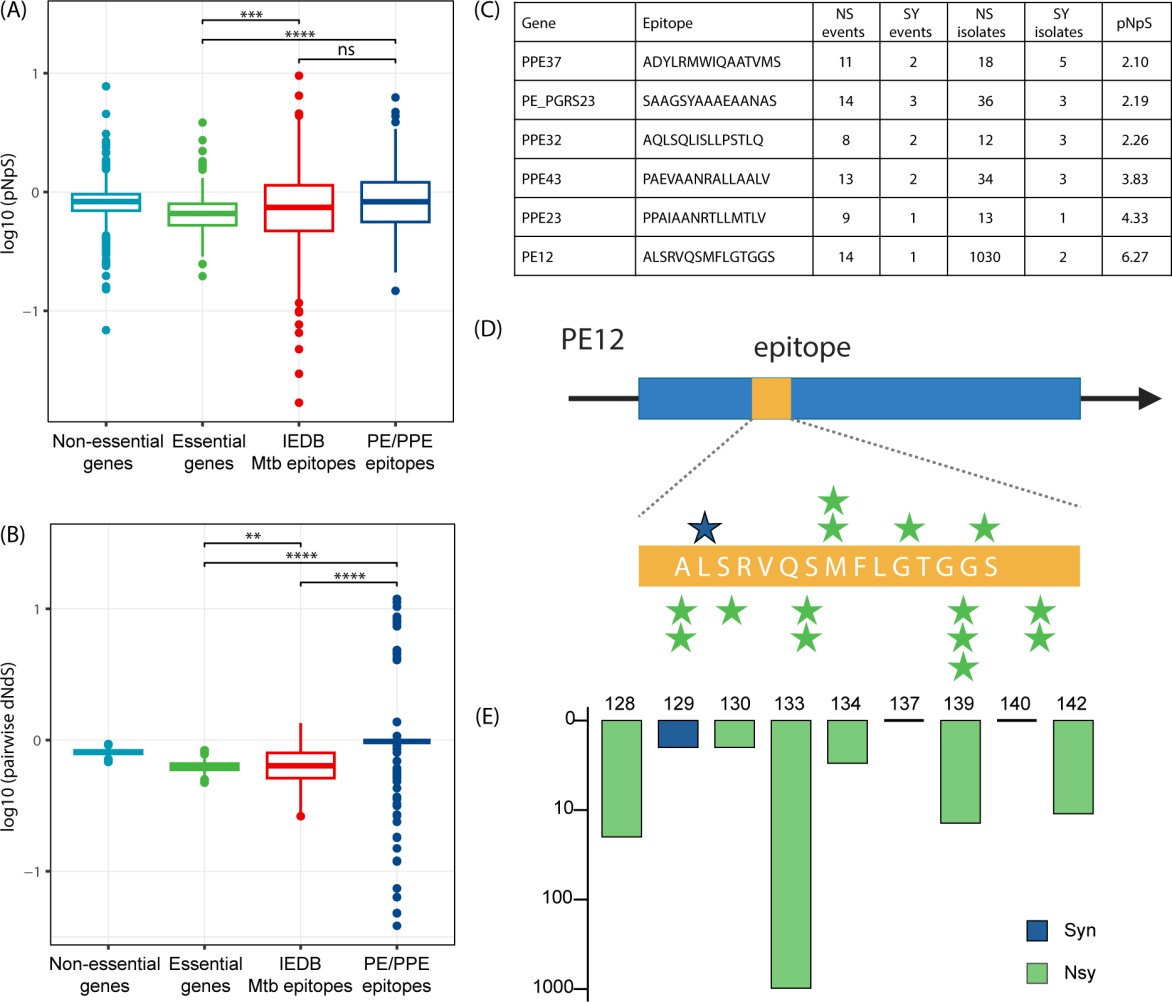
Selective pressure on PE/PPE Epitopes. (**A**) Comparison of *pNpS* ratios between epitopes from PPE genes and other genomic regions. (**B**) Pairwise dN/dS ratios showing elevated selective pressure in PPE epitopes relative to epitopes from other genomic regions and essential genes. (**C**) PE/PPE epitopes exhibiting high *pNpS* values, highlighting epitopes under positive selection. (**D**) Representation of mutation sites within the PE12 epitope region, emphasizing positions with multiple independent mutation events. (**E**) Distribution of the number of isolates carrying different codon mutations within the PE12 epitope region. Pairwise comparisons were conducted using the Wilcoxon rank-sum test. Statistical significance is indicated by asterisks (*P* < 0.05); "ns" represents non-significant differences (*P* ≥ 0.05).

Antigen recognition of Mtb is a crucial component of the host immune response and a key driver in the evolution of Mtb antigenic epitopes (20, 21). Research on epitopes outside of the PE/PPE gene family has suggested that most epitopes are highly conserved (20, 21), but very few studies have examined epitopes within the PE/PPE gene family. When we compared the epitopes in PE/PPE genes with epitopes in other genomic regions, there were no significant differences in the *pNpS* values (Fig. 4A). However, in contrast to previous studies, the *pNpS* ratios for both PE/PPE and non-PE/PPE epitopes were higher than the *pNpS* ratios for essential genes. To validate this finding, we calculated pairwise dN/dS, which assesses the ratio of NS to SY and also considers the allele frequency of NS mutations (Materials and Methods) (25). The pairwise comparison confirmed that both PE/PPE and non-PE/PPE epitopes had higher dN/dS ratios than essential genes (Fig. 4B) and also found that PE/PPE epitopes had significantly higher dN/dS than non-PE/PPE epitopes, suggesting that PE/PPE epitopes could be under relaxed purifying selection. Epitopes with high *pNpS* ratios were found in PPE23 (3.83) and PPE43 (4.33) (Fig. 4C), but the highest was in PE12, which had a *pNpS* of 6.27 (14 NS, 1 SY) and contained five sites with more than two convergent mutation events (Fig. 4D). The Q133H mutation arose independently in the ancestors of Lineage 6 and *M. bovis*, and also the ancestor of Lineage 4.9, with 960 and 18 isolates, respectively, carrying this mutation (Fig. 4E). Within the PE/PPE gene family, however, the *pNpS* of the epitopes was not significantly different from the *pNpS* in the non-epitope regions (Fig. S3).

### Deciphering selective forces on PE/PPE genes across evolutionary, functional, and structural dimensions

It is thought that the PE/PPE gene family originated from the ancestral PE5-PPE4 gene pair within the ESX-3 locus and subsequently expanded through duplication events. Based on phylogenetic analysis of the PE/PPE domain sequences, PE/PPE genes can be divided into groups II (PPW family), III, IV (SVP family), and V (MPTR family), and these groupings correspond well with the evolutionary origin order of the PE/PPE genes. For instance, group IV (SVP family) is believed to have originated from the common ancestor of all slow-growing mycobacteria, whereas group V (the MPTR family) is thought to have originated from the common ancestor of phylogenetically related, slow-growing mycobacteria *M. gordonae*, Mtb, and *M. kansasii* (18). Thus, the temporal sequence of the origin of these groups was, from earlier to later, II (PPW), III, IV (SVP), and V (MPTR). We hypothesized that the most recently evolved PE/PPE genes might still be under adaptive selection and thus might exhibit different selective pressures compared to those that evolved earlier. We compared the *pNpS* values of PE/PPE genes from each of these groups but found no significant differences in selection pressure (Fig. S4). It thus appears that even the most recently emerged PE/PPE genes have, as a group, become genomically localized and tend to be conserved in Mtb strains.

In addition to analyzing the PE/PPE genes based on their evolutionary origins, we also investigated natural selection from the perspective of functional importance. Given the critical biological functions of the ESX secretion system, we hypothesized that the PE and PPE proteins within the ESX loci would be more conserved. PE and PPE protein pairs, such as PE25-PPE41 and PE5-PPE4, form heterodimers that are secreted out of the bacteria, and the genes encoding these dimer-forming proteins are generally adjacent to each other within the genome. Accordingly, we classified the PE and PPE genes into three groups: those within the five ESX loci; those occurring in pairs; and those occurring as single genes. We then compared the *pNpS* ratios among these groups and found that the PPE genes within ESX loci have a significantly lower *pNpS* ratio compared to the other two groups and also have NS mutation burdens that are lower, although not statistically different (Fig. S5). No similar trend was observed in the PE genes.

The PE and PPE domains form a helix-turn-helix hairpin structure, typically assembling into heterodimers that spatially align the WxG and YxxxD/E motifs to create a composite structure recognized by the Type VII secretion system. Therefore, the PE and PPE domains, along with their associated motifs, are expected to be highly conserved. We analyzed the diversity and natural selection acting on these domains and motifs and found, surprisingly, no significant difference in *pNpS* ratios between the PE and PPE domains and their respective C-termini (paired Wilcoxon test) (Fig. S6A). The average *pNpS* for the PPE domain and the PPE C-terminus were 0.88 (95% CI 0.81–0.94) and 0.89 (95% CI 0.83–0.95), respectively. For the PE domain and the PE C-terminus, the average *pNpS* values were 0.88 (95% CI 0.82–0.93) and 0.94 (95% CI 0.85–1.0), respectively. Given the significant difference in high-quality coverage between the PE/PPE domains and the C-terminal regions (Fig. S1), we stratified the 116 genes into two groups based on their overall coverage: a low coverage group (50%–80% high-quality region; Fig. S6B) and a high coverage group (>80% high-quality region; Fig. S6C). Within each group, we compared the pNpS values between the PE/PPE domains and the C-termini. In both cases, no significant differences were observed. Although there is no significant difference between the domains and the C-termini when all PPE genes are considered together, certain PPE genes exhibit higher *pNpS* ratios in the PPE domain compared to the C-terminus. For example, in PE_PGRS34 (high-quality region proportion: 0.79), the *pNpS* ratio for the PE domain is 1.38, compared to 0.82 for the PGRS domain.

## DISCUSSION

In this study, we investigated the adaptive evolution of the PE/PPE gene family in response to host immune and antibiotic pressures using WGS data from approximately 50,000 Mtb isolates. This analysis was performed at both the individual gene level across sequence domains, PE/PPE subfamilies, and Mtb lineages. We found that the PE/PPE gene family exhibits higher mutational diversity and *pNpS* ratios compared to other gene categories, suggesting a relaxed purifying selection. However, 12 genes and multiple epitopes in genes thought to be involved in nutrient uptake, metal ion transport, and lipase activity were under positive selection. The seven genes for which the selection was associated with antibiotic resistance included PPE51—a recently characterized transporter protein that may compensate for the evolutionary loss of porins in Mtb (3, 26). Additionally, we identified 13 highly conserved PE/PPE genes (pN/pS < 0.7) as potential candidates for future vaccine development.

Due to the repetitive and highly similar sequences within the PE/PPE gene family, analyzing these genomic regions using short-read sequencing has been particularly challenging (27). Consequently, most Mtb genomic studies have excluded PE/PPE genes, seriously limiting our understanding of sequence diversity and evolutionary signatures within this gene family (12, 13). While some recent studies have used third-generation, long-read sequencing to investigate PE/PPE genes (1, 11), the high cost of this technology limits its widespread application, especially for population-based studies involving thousands of Mtb isolates. Fortunately, however, a recent genome masking scheme has shown that many PE/PPE genes can be reliably analyzed using short-read sequencing data (14), which has enabled the reanalysis of publicly available short-read sequencing data and facilitated the exploration of evolutionary signatures in the PE/PPE genes. By employing this gene masking scheme, we could analyze ~54% of PE/PPE gene regions in ~50,000 Mtb genomes to obtain the first population-scale view of the genetic diversity of this gene family.

We identified seven PE/PPE genes that may be associated with drug resistance. These drug resistance-associated genes appear to be enriched in proteins involved in outer membrane metal ion transport and nutrient uptake, such as *PPE51*, *PE15*, and *PE20 (3, 26, 28*). The *PPE51–PE19* pair is believed to function as a porin-like complex involved in the uptake of glycerol, glucose, and phosphate (3, 26). Although several other PE/PPE pairs have been implicated in nutrient transport across the outer membrane, their contribution to drug resistance is unclear. Wang et al. found that the deletion of *PPE51* in Mtb conferred resistance to small molecules, presumably by blocking the uptake of growth-inhibiting compounds. Here, we found that *PPE51* was under positive selection, but only after the bacteria had acquired mutations in *katG* and *rpoB* to become multidrug-resistant. We found that 43/48 of the mutations in *PPE51* were frameshift indels or premature-stop mutations, suggesting that the selective pressure favors loss of *PPE51* function. Many of these strains had also accumulated mutations in other drug resistance genes, such as *gyrA, rrs*, and even *mmpR5*, before acquiring the *PPE51* mutations. Enrichment for nonsynonymous *PPE51* mutations was most significant in strains resistant to six to eight anti-TB drugs, suggesting that *PPE51* mutations arise at a very late stage of drug resistance evolution. This raises the possibility that the loss of *PPE51* may be involved in resistance to recently introduced drugs, such as bedaquiline (BDQ) or linezolid (LZD), which are frequently prescribed after resistance to first- and second-line agents has emerged. We examined 105 BDQ-resistant strains from the WHO-Catalogue data set and found that only two carried PPE51 mutations, limiting our ability to assess the statistical significance. Alternatively, PPE51 mutations may contribute to the evolution of late-stage drug resistance by restoring fitness in MDR/XDR strains. Functional studies of genes that are selectively mutated in MDR backgrounds require the manipulation of MDR-TB strains, which is prohibited in many BSL-3 laboratories. Therefore, broader population-level data from clinical isolates will be essential for deciphering the role of *PPE51* in the evolution of Mtb drug resistance.

Our results imply that selective mutational pressures are acting on PE/PPE genes involved in nutrient and metal ion transport to remodel Mtb’s metabolic interface with the host. For example, PE15, a substrate of the ESX-3 secretion system, forms a functional complex with PPE20 and is implicated in Ca²^+^ transport; and PPE31 may be involved in Mg²^+^ uptake (3, 26, 28). Similarly, PPE36 and PPE37, which are essential for heme iron acquisition, showed elevated *pNpS* values in drug-resistant isolates, suggesting ongoing adaptation to iron availability during the evolution of drug resistance (29, 30). The association between metal transport and drug resistance may stem from shared regulatory pathways or cross-resistance mechanisms. In other bacteria, such as *E. coli* and *S. aureus*, metal ion stress induces overexpression of efflux pumps that can also expel antibiotics (31, 32). A similar mechanism may exist in Mtb, in which zinc transporter Rv3270 and nickel/cobalt transporter Rv2856 have been associated with enhanced drug efflux (33, 34). Taken together, these findings suggest that antibiotic exposure may drive Mtb to adapt not only through classical drug resistance pathways but also by altering fundamental systems involved in nutrient and metal acquisition to maintain homeostasis and fitness in hostile environments.

Our analysis also identified evidence of diversifying selection in multiple PE/PPE-derived T-cell epitopes, revising the long-held view that Mtb epitopes are evolutionarily hyperconserved (20, 21). Notably, epitopes in genes such as PE12 and PPE23 displayed elevated *pNpS* and dN/dS ratios, with multiple convergent nonsynonymous mutations arising independently across different Mtb lineages. This suggests that some PE/PPE antigens may be evolving to evade host T-cell recognition, which could have potentially important implications for TB vaccine design. Many subunit vaccine candidates, including ID93 and M72/AS01E (2, 7), incorporate PE/PPE proteins because of their immunogenicity and location on the outer surface of the bacteria. However, the epitope variation we observed raises questions about antigenic stability across the population of circulating Mtb strains. It will be important to evaluate whether this epitope variability could reduce vaccine efficacy in genetically diverse populations or promote immune escape following vaccine rollout.

Based on established phylogenetic groupings (e.g., MPTR, SVP, PPW) of the PE/PPE gene family, we hypothesized that recently duplicated genes would exhibit higher *pNpS* ratios, reflecting ongoing adaptation. However, our analysis revealed no significant differences in the selection pressures on these subgroups, suggesting that most PE/PPE genes, regardless of their evolutionary age, have undergone functional stabilization. This observation implies that PE/PPE genes are not merely products of genetic drift or random expansion but have likely acquired species-specific biological roles that are now maintained under purifying selection. This reinforces the emerging view that many PE/PPE proteins are involved in specialized and conserved functions related to host interactions, nutrient acquisition, or immune modulation (3–7).

*Limitations and future directions*. Despite analyzing approximately 54% of the PE/PPE genomic content using short-read data, limitations remain in resolving regions with extreme repetitiveness or high GC content, such as PGRS-rich sequences and tandem duplications. As a result, the diversity and evolutionary dynamics of some PE/PPE genes—especially those with known immunomodulatory functions—may be underrepresented in our study. Future studies employing long-read sequencing technologies, such as PacBio HiFi or Oxford Nanopore (35, 36), could complement our findings and expand coverage to previously inaccessible regions. Additionally, functional validation of positively selected genes and epitopes—through gene knockouts, host-pathogen interaction assays, and animal models—will be essential to determine their precise roles in Mtb adaptation, transmission, and drug resistance. Together, these approaches will provide a more complete picture of how PE/PPE genes contribute to the evolutionary success of Mtb.

In summary, this study reanalyzed approximately 54% of the PE/PPE gene sequences in WGS data from ~50,000 Mtb isolates, providing a population-scale view of their genetic diversity and adaptive evolution. We identified several members of this gene family subject to positive selection, including genes linked to host immune interactions or antibiotic resistance, thereby providing new candidate genes and novel directions for future research into resistance mechanisms and Mtb pathogenicity.

## MATERIALS AND METHODS

### Quality control and preprocessing of WGS data

We previously collected WGS data from 51,229 Mtb isolates across 209 studies deposited in the Sequence Read Archive (SRA) (15). Raw sequencing data were downloaded using the prefetch command from the SRA toolkit, followed by extraction of FASTQ files using fastq-dump. To ensure high-quality sequencing data, isolates were filtered based on the following criteria: average sequencing depth ≥20×; mapping rate ≥90%; and genome coverage ≥99% at ≥10× depth. Species-level confirmation was performed using Kraken2 (37), and only samples with ≥95% of reads classified as Mtb were retained. Lineage assignment and detection of mixed infections were conducted using fastlin (38); samples identified as mixed infections were excluded from downstream analyses.

### PE/PPE high-quality regions

The Mtb genome low-quality region BED file was obtained from the Supplementary material of Maximillian Marin’s study (14). High-quality regions were defined by excluding low-quality segments from this BED file and subsequently visualized using the R gggenome package for PE/PPE genes. The proportion and length of high-quality regions were calculated for each gene.

### Mutation detection

#### Fixed mutation detection

Adapters and low-quality bases were trimmed from FASTQ reads using Sickle, retaining reads longer than 30 bases with an average quality score above 20. The reads were aligned to the Mtb ancestral reference genome (39) using Bowtie2 (v2.2.9) (40). Samtools (v1.3.1) was used to calculate sequencing depth and coverage, and samples with an average depth below 20× or genome coverage below 90% were excluded (41). Single-nucleotide variants (SNVs) were detected using VarScan (v2.3.9) with the following criteria: mapping quality >30; depth >10; strand bias ratio >0.8; and frequency >0.9. SNVs located in the Mtb genome’s low-quality regions were filtered out (42). Small indels were identified using VarScan (v2.3.9) with the mpileup2indel function.

#### Unfix mutation detection

Unfixed SNVs were identified using VarScan (v2.3.9) from the BAM file (42). SNVs with a frequency between 1% and 90%, supported by at least three reads (with at least one read from each strand), were detected with the strand bias filter enabled. A previously validated pipeline was employed to filter out false positives (43). First, mpileup files were generated using the samtools mpileup function to identify the locations of mutant alleles in the sequencing reads, and unfixed SNVs with more than 50% of supporting alleles enriched in the terminal regions of reads were excluded. Second, the sequencing depth of sites with unfixed SNPs had to be within 50%–150% of the isolate’s average depth to eliminate false positives due to insertions, deletions, or duplications. Third, all unfixed SNPs mapped to the Mtb genome low-quality regions were excluded. Lastly, unfixed SNPs that occurred repeatedly with similar mutational frequencies (suggestive of multi-locus mapping) were also removed.

### Mutation events detection and annotation

Samples were categorized into 25 sublineages based on established Mtb sublineage-defining SNPs. The identification of mutation events relied on the phylogenetic tree structure. Therefore, we first excluded SNVs located in repetitive regions of the Mtb genome and in low-confidence PE/PPE regions identified by a recent study (14), retaining only SNVs in high-quality PE/PPE regions. The filtered SNVs were then combined into a non-redundant consensus list. Using the mpileup2cns function in VarScan, mutations at each site in the consensus list were recalled. Sites with missing calls in more than 5% of samples were removed from the recalled mutations. The remaining sites were used to create a concatenated alignment for phylogenetic tree construction. Phylogenetic trees and ancestral sequence inference for each phylogeny node were performed using IQ-TREE2, with the Mtb ancestor sequence set as the root, using the GTR nucleotide substitution model, and a bootstrap replicate count of 1,000 (17). The iTOL tool was used for tree visualization and annotation (44).

Starting from the root, mutation events at each node of the phylogenetic tree were inferred sequentially to the tree leaves. Mutation events at each node were identified by comparing the ancestral sequences of the node and its parent. If the sequences of the node and its parent were identical, it was concluded that no mutations occurred at that node. This process was implemented using custom scripts. The mutation events were annotated using custom scripts, including information such as the affected gene, codon position, and mutation type.

### *pNpS* and mutation burden calculation

The *pNpS* value for each PE/PPE gene was calculated using a previously established script (43). The mutation burden for each gene was determined by normalizing the gene length and total mutation events as follows:


$$Di=\frac{Mi}{N\;*\;Li}$$

where *Di* represents the mutation burden of gene *i*, and *Mi* represents the number of mutation events in gene *i. N* is the total number of mutation events, and *Li* is the length of the high-quality region of gene *i*. These calculations were performed for each sublineage as well as for the entire data set.

### Drug resistance-associated gene analysis

To identify genes associated with drug resistance, the data set was divided into drug-susceptible and drug-resistant groups based on molecular drug susceptibility testing (DST) results. Molecular DST for each isolate was performed using the WHO Drug Resistance-Associated Mutation Catalogue, 1st edition (https://www.who.int/publications/i/item/9789240028173). Isolates carrying mutations with a final confidence grading of “Assoc w R” were classified as drug resistant, while those without these mutations were identified as drug-susceptible. For each data set, the *pNpS* value for each gene was calculated. The top drug resistance-associated genes were selected based on the following criteria: (i) genes with a high confidence region ratio greater than or equal to 0.5; (ii) the number of mutation events in drug-resistant isolates exceeding 30; (iii) a *pNpS* value in drug-susceptible isolates less than 1.05; (iv) a delta value (the *pNpS* value in drug-resistant isolates minus the *pNpS* value in drug-susceptible isolates) greater than 0.4; and (v) removal of pseudogenes and possible truncated genes, including PE21, PE_PGRS12, PE_PGRS40, PPE50, PPE67, PPE7.

In addition to the *pNpS* calculations, we analyzed the distribution of NS mutations in drug-resistant and drug-susceptible isolates. The R chi-squared test was employed to identify genes with significantly enriched NS mutations in drug-resistant isolates.

### Epitope identification

Epitope sequences were downloaded from the IEDB (https://www.iedb.org/result_v3.php?cookie_id=8cc14d&active_tab=Epitopes) in March 2023. The selection of epitopes was conducted according to the screening criteria outlined in (21). Specifically, we accepted only epitopes fulfilling the following criteria: linear peptides; M. tuberculosis complex; positive assays only; T-cell assays, any MHC restriction; human host; association with any disease; and any reference type. This process resulted in the identification of 2,141 epitopes. Epitopes located within PE/PPE genes were designated as PE/PPE epitopes, while the remaining epitopes were labeled as IEDB Mtb epitopes. The *pNpS* values were calculated for each epitope individually. Epitopes with more than 10 mutation events and a *pNpS* value greater than 2 were selected as the top positively selected epitopes, as shown in Fig. 4C.

### Pairwise dN/dS calculation

Due to the extensive computational burden associated with calculating pairwise dN/dS values for nearly 50,000 isolates, we randomly selected 5,000 isolates. The genes from each of these isolates were categorized into one of four groups: essential; non-essential; IEDB epitopes; and PE/PPE epitopes. After replacing the reference sequence with the SNVs from each isolate, we concatenated the genes within each group to form a concatenated sequence. Subsequently, the concatenated sequences from the 5,000 isolates were merged into a single file to serve as the input FASTA for pairwise dN/dS. The pairwise dN/dS values for essential, non-essential, IEDB epitopes, and PE/PPE epitopes were calculated using the kaks function from the R package seqinr. Analysis code relevant to this study is available at https://github.com/irongump/MTB_PEPPE.

## SUPPLEMENTAL MATERIAL

Fig. S1 msystems.00898-25-s0001.tif Proportion of high-quality sequence regions located within the PE/PPE domains compared to the C-terminus or PGRS regions.

Fig. S2 msystems.00898-25-s0002.tif Comparison of mutation burdens, and pNpS ratios among PE/PPE genes, essential genes, nonessential genes, and other functional gene categories.

Fig. S3 msystems.00898-25-s0003.tif Comparison of pNpS ratios between epitope and non-epitope regions within the same PE or PPE genes.

Fig. S4 msystems.00898-25-s0004.tif Comparative analysis of pNpS ratios and mutation burdens among different evolutionary subgroups of PPE genes and PE genes.

Fig. S5 msystems.00898-25-s0005.tif Comparison of pNpS ratios and mutation burdens among PE/PPE genes grouped by genomic context.

Fig. S6 msystems.00898-25-s0006.tif Comparison of selective pressure (pNpS) between conserved PE/PPE domains and their corresponding C-terminal regions.

Legends msystems.00898-25-s0007.docx Supplemental material legends.

Table S1 msystems.00898-25-s0008.xlsx SRA accession numbers.

Table S2 msystems.00898-25-s0009.xlsx PE and PPE gene genome postions and high quality region length and proportion.

Table S3 msystems.00898-25-s0010.xlsx Bed file of high quality regions.

ASM does not own the copyrights to Supplemental Material that may be linked to, or accessed through, an article. The authors have granted ASM a non-exclusive, world-wide license to publish the Supplemental Material files. Please contact the corresponding author directly for reuse.
