# Supplementary figures and images for: Ongoing evolution of PE/PPE genes in Mycobacterium tuberculosis associated with drug resistance and host immune response
Source: mSystems. 2025 Sep 22;10(10):e00898-25. doi: 10.1128/msystems.00898-25 (PMC12542625; doi:10.1128/msystems.00898-25)

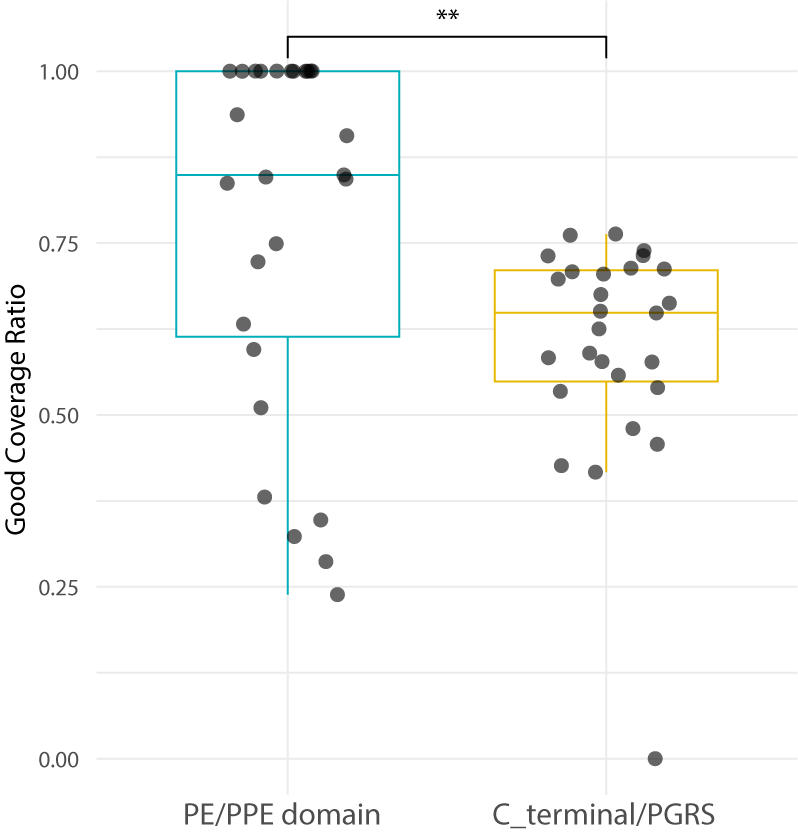

Supplement: Fig. S1 — Proportion of high-quality sequence regions located within the PE/PPE domains compared to the C-terminus or PGRS regions. [file msystems.00898-25-s0001.tif]

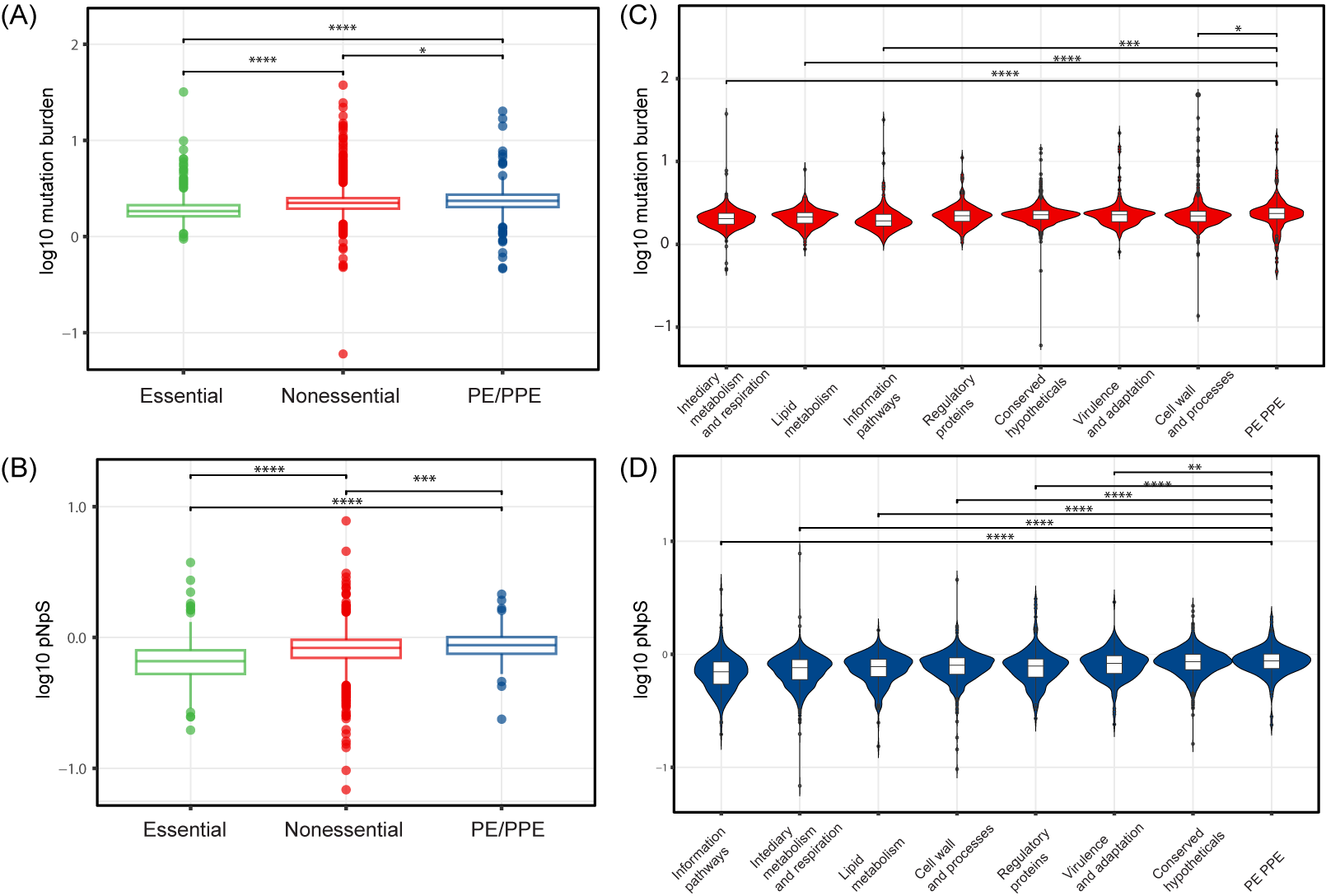

Supplement: Fig. S2 — Comparison of mutation burdens, and pNpS ratios among PE/PPE genes, essential genes, nonessential genes, and other functional gene categories. [file msystems.00898-25-s0002.tif]

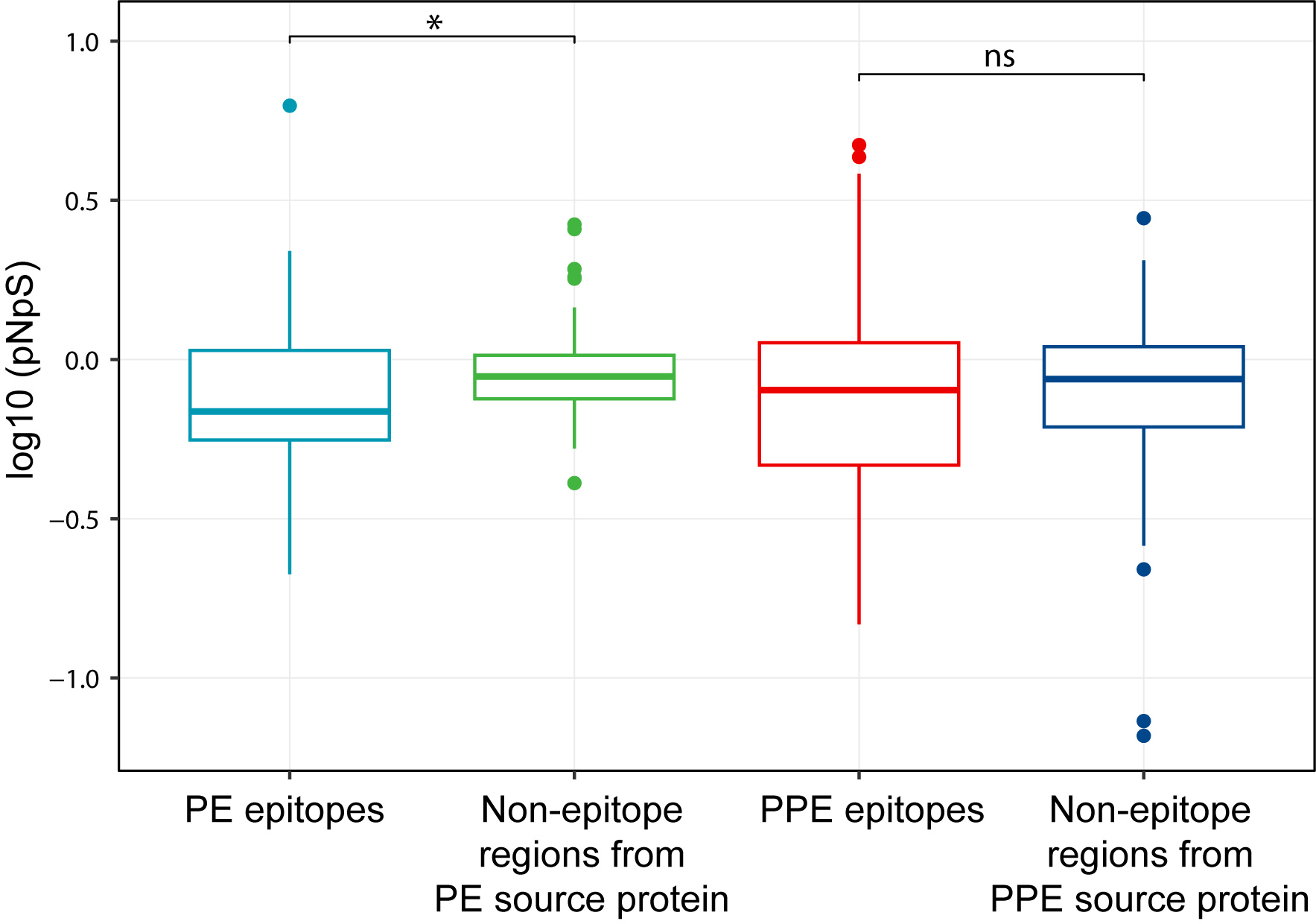

Supplement: Fig. S3 — Comparison of pNpS ratios between epitope and non-epitope regions within the same PE or PPE genes. [file msystems.00898-25-s0003.tif]

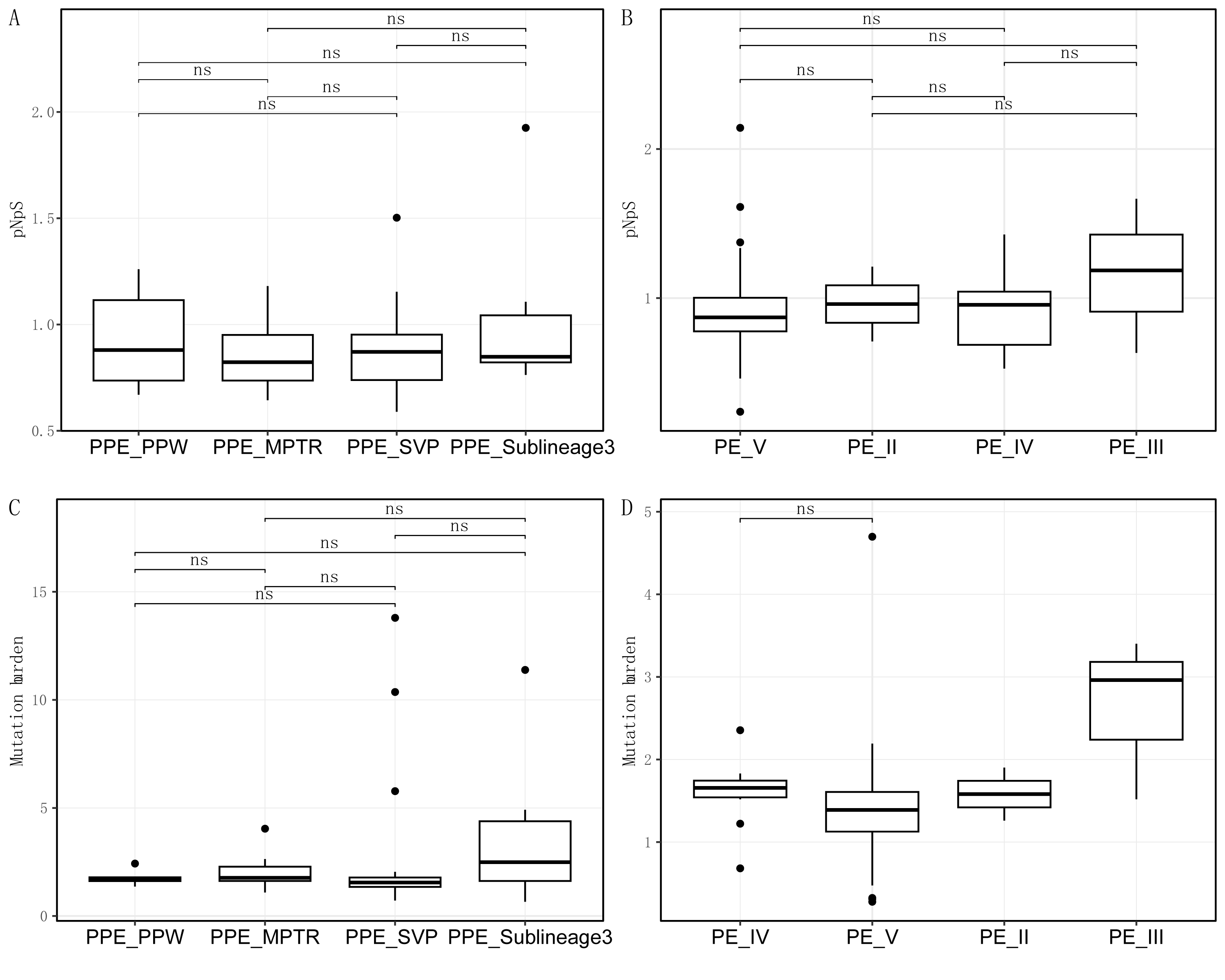

Supplement: Fig. S4 — Comparative analysis of pNpS ratios and mutation burdens among different evolutionary subgroups of PPE genes and PE genes. [file msystems.00898-25-s0004.tif]

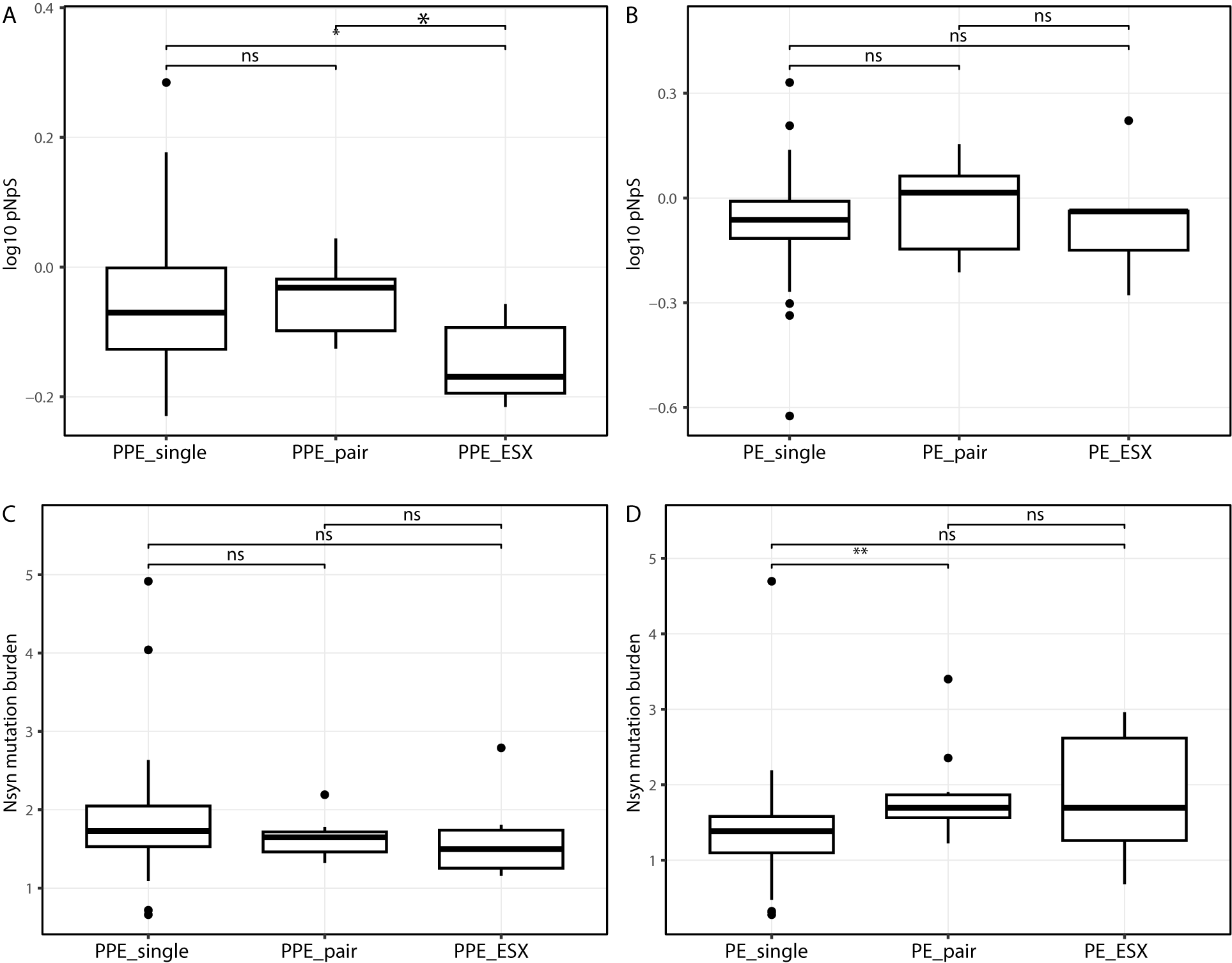

Supplement: Fig. S5 — Comparison of pNpS ratios and mutation burdens among PE/PPE genes grouped by genomic context. [file msystems.00898-25-s0005.tif]

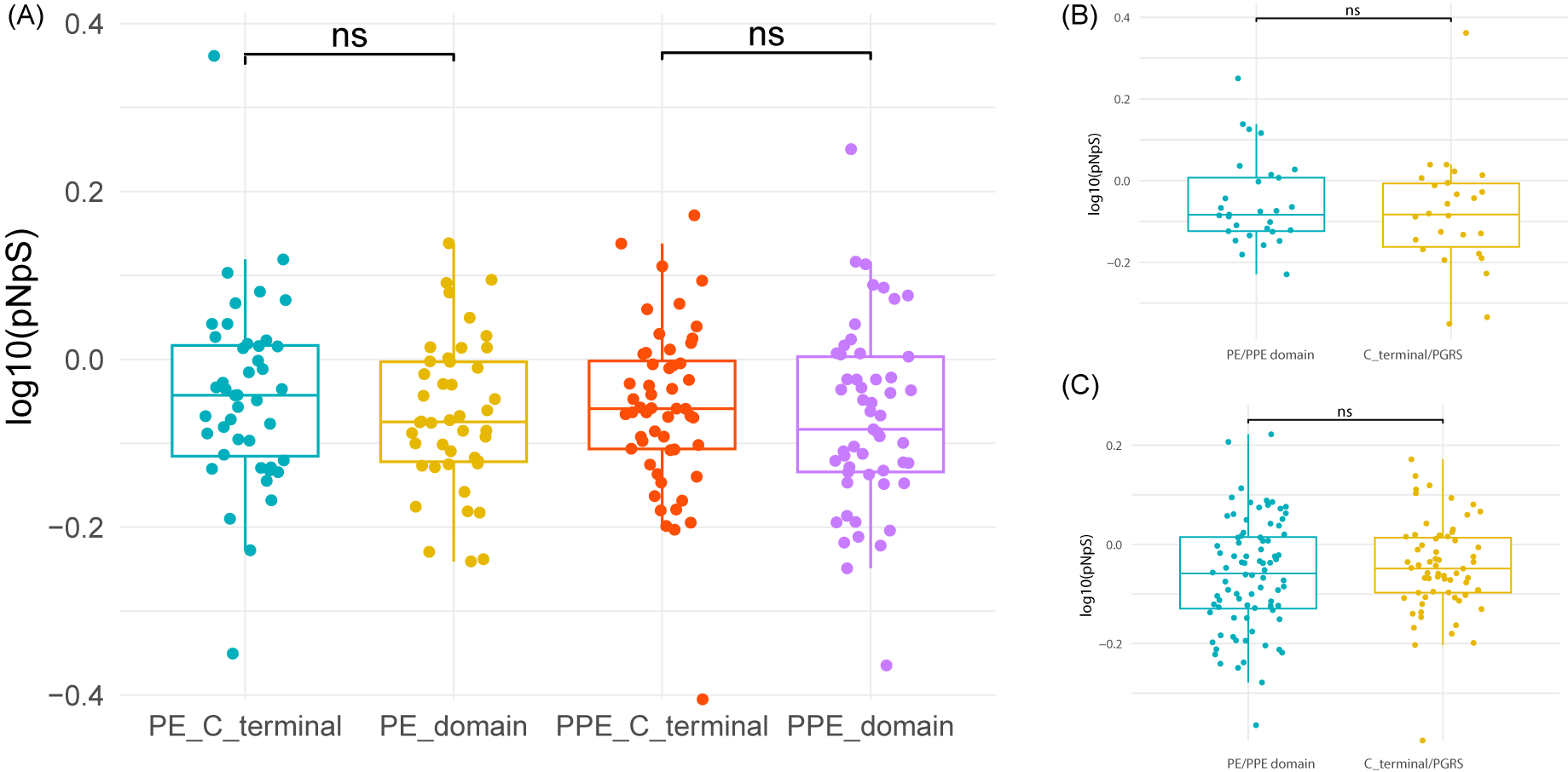

Supplement: Fig. S6 — Comparison of selective pressure (pNpS) between conserved PE/PPE domains and their corresponding C-terminal regions. [file msystems.00898-25-s0006.tif]
