# Supplementary material for: Ongoing evolution of PE/PPE genes in Mycobacterium tuberculosis associated with drug resistance and host immune response
Source: mSystems. 2025 Sep 22;10(10):e00898-25. doi: 10.1128/msystems.00898-25 (PMC12542625; doi:10.1128/msystems.00898-25)
Supplement: Legends — Supplemental material legends. [file msystems.00898-25-s0007.docx]

**Fig S1**. Proportion of high-quality sequence regions located within the PE/PPE domains compared to the C-terminus or PGRS regions. Pairwise comparisons were conducted using the Wilcoxon rank-sum test. Statistical significance is indicated by asterisks (*P* < 0.05).

**Fig S2**. Comparison of mutation burdens (A), and *pNpS* ratios (B) among PE/PPE genes, essential genes, nonessential genes, and other functional gene categories (C and D). The y-axis values for both *pNpS* and mutation burden are shown after log₁₀ transformation. Pairwise comparisons were conducted using the Wilcoxon rank-sum test. Statistical significance is indicated by asterisks (*P* < 0.05).

**Fig S3.** Comparison of *pNpS* ratios between epitope and non-epitope regions within the same PE or PPE genes. Pairwise comparisons were conducted using the Wilcoxon rank-sum test. Statistical significance is indicated by asterisks (P < 0.05); "ns" represents non-significant differences (P ≥ 0.05).

**Fig S4.** Comparative analysis of pNpS ratios and mutation burdens among different evolutionary subgroups of PPE genes (A & C) and PE genes (B & D). Pairwise comparisons were conducted using the Wilcoxon rank-sum test. "ns" represents non-significant differences (P ≥ 0.05).

**Fig S5.** Comparison of *pNpS* ratios (A & B) and mutation burdens (C & D) among PE/PPE genes grouped by genomic context: ESX locus-associated; paired; or single genes. Pairwise comparisons were conducted using the Wilcoxon rank-sum test. Statistical significance is indicated by asterisks (*P* < 0.05); "ns" represents non-significant differences (*P* ≥ 0.05).

**Fig S6**. Comparison of selective pressure (pNpS) between conserved PE/PPE domains and their corresponding C-terminal regions. (A) Results from all 116 genes included in the analysis. (B) Subset of genes with low coverage (50%–80% high-quality regions). (C) Subset of genes with high coverage (>80% high-quality regions). Pairwise comparisons were conducted using the Wilcoxon rank-sum test. "ns" represents non-significant differences (P ≥ 0.05).

**Table S1.** SRA accession numbers used in this study.

**Table S2.** PE and PPE genes genome positions and high quality region length and proportion.

**Table S3.** High quality region’s start and end positions.
